# Patent Bridge Work

**Published:** 1885-05

**Authors:** 


### PATENT BRIDGE WORK.

We publish in this number a communication from the International Tooth Crown Co., in answer to one from Dr. Dwinelle in the number for October last. Having given place to the one, we cannot refuse the other. Concerning the facts in the case we have no personal knowledge. Our readers must judge for themselves as between Dr. Dwinelle and Sheffield. But one thing we wish to say very emphatically: this communication closes out the discussion of the tooth crown patents in this journal. The subject is becoming too odorous.

